# “When the going gets tough, the tough get going”: Motivation towards closure and effort investment in the performance of cognitive tasks

**DOI:** 10.1007/s11031-017-9613-y

**Published:** 2017-04-19

**Authors:** Sindhuja Sankaran, Ewa Szumowska, Małgorzata Kossowska

**Affiliations:** Institute of Psychology, Jagiellonian University, Ingardena Str. 6, 30-060 Krakow, Poland

**Keywords:** Need for closure, Effortful and effortless means, Goal importance, Cognitive energetics theory

## Abstract

Previous studies have demonstrated that the need for closure (NFC), which refers to an individual’s aversion toward uncertainty and the desire to quickly reduce it, leads to reluctance to invest effort in judgments and decision making. However, we argue that NFC may lead to either an increase or a decrease in effort depending on the availability of easy vs. difficult means to achieve closure and perceived importance of the task goal. We found that when closure could be achieved via both less and more demanding means, NFC was associated with decreased effort unless the task was perceived as important (Study 1). However, when attaining closure was possible via demanding means only, NFC was associated with increased effort, regardless of the task importance (Study 2). Moreover, NFC was related to choosing a more instrumental strategy for the goal of closure, even if this strategy required effort (Study 3). The results are discussed in the light of cognitive energetics theory.

**Electronic supplementary material:**

The online version of this article (doi:10.1007/s11031-017-9613-y) contains supplementary material, which is available to authorized users.

## Introduction

Previous studies demonstrated that the need for closure (NFC), referring to an individual’s aversion toward uncertainty and the desire to avoid or quickly reduce it, leads to effortless processing styles, i.e., category-based, nonsystematic and heuristic (Kruglanski 2004, for overview). Thus, the typical effects of NFC, such as a less extensive search for information, limited number of generated hypotheses, primacy effect in impression formation, numerical anchoring, or preference for quick decision-making (Kruglanski and Webster 1996) were usually interpreted as a general reluctance to invest effort in judgments and decision making. However, we argue that NFC may lead to either an increase or a decrease in effort investment depending on whether closure is possible to achieve via less or more cognitively demanding means and on perceived importance of the task goal. Thus, we claim that NFC is associated with processing strategies adapted to salient goals and inner states. Our studies contribute to the discussion on the role of effort investment in social cognition in general (see Kruglanski et al. 2012) and to the discussion on the need for closure theory more specifically (Roets et al. 2015).

## NFC and increased mobilization of effort

Although the bulk of the NFC research has demonstrated that NFC is linked to a preference for effortless strategies (Kruglanski and Webster 1996), there are some studies showing the opposite relationship, i.e., that under certain circumstances high NFC also leads to more effortful cognitive strategies (see Roets et al. 2015, for overview of NFC research supporting both claims). For instance, high NFC induced a heightened level of information processing when initial confidence was low, as opposed to when initial confidence was high (Kruglanski et al. 1991), and high NFC was found to be related to a greater openness to persuasion when an informational base for an opinion was absent (Kruglanski et al. 1993). Also, Van Hiel and Mervielde (2002, Experiment 4) have shown that high NFC intensifies information acquisition when ambiguous information enters the judgmental process. Likewise, Klein and Webster (2000) demonstrated that individuals high in NFC processed a message systematically when heuristic cues were not available. Also, Vermeir et al. (2002) tested participants in a series of low involvement decisions wherein they asked participants to choose between brands of unfamiliar products, so that prior knowledge could not be relied upon. They found that high (as compared to low) NFC individuals searched for significantly more information before their opinion was crystallized, but not thereafter. In a similar vein, Houghton and Grewal (2000) showed that high levels of NFC led to less extensive information search in a consumer choice paradigm, but only when participants had a previously formed attitude about the product.

In sum, all of these results suggest that, although generally not prone to invest cognitive effort, individuals high in NFC are motivated to do so in some instances, i.e., when closure cannot be attained with the use of undemanding, effortless means. In other words, one might infer from this that given a choice, individuals high in NFC would choose the effortless, i.e., easiest and quickest, way to attain closure. However, if this effortless route is made unavailable, they are willing to invest effort to attain closure.

## Goal importance

Cognitive energetic theory (CET) provides insight into the mechanisms underlying the selection of the cognitive effort level for a given task (Kruglanski et al. 2012). CET, which builds on previous theories such motivation intensity theory (Brehm and Self 1989; Wright and Brehm 1984) and the lay epistemic theory (Kruglanski 1989), posits that at the moment of choice, there are forces driving cognitive effort (e.g., goal importance and resource availability), and forces restraining cognitive effort (e.g., task demands and the personal tendency to conserve cognitive resources, e.g., NFC). The authors argue that goal importance is determined by the value attached to the goal, or its desirability, and its attainability, or the expectancy of its completion (see also Atkinson and Birch 1970; Brehm and Self 1989). The more important a given goal is to a person, the more likely s/he is to invest effort to attain that goal (Kruglanski et al. 2012).

However, certain goals are more important for some people than for others. It has been argued that dealing with uncertainty is a main cognitive goal driving the behavior of individuals high in NFC (Kruglanski 1990). Thus, we suggest that, due to their intolerance of uncertainty, high NFC individuals have a stronger motivation to reduce this state than low NFC individuals, and thereby perceive attaining closure as an important goal. Given the availability of cognitive resources, with an increase in the importance of the goal to achieve closure, high NFC individuals are more willing to do whatever is necessary to achieve their goal, that is, to gain certainty.

In a task performance situation, this high NFC individuals’ desire for certainty manifests itself in a greater striving to gain certainty that the task requirements have been or will be met (see also Chiu et al. 2000; Fu et al. 2007; Jia et al. 2014; Szumowska and Kossowska 2017). And, it depends on the task whether this certainty can be gained with the use of effortful or effortless means. If only effortful means provide such certainty, NFC should lead to more effort. In a performance task, in which the goal is to get the best score (herein referred to as the task goal), the closure goal and the task goal converge. So, two otherwise independent goals, goal to perform well on the task (the task goal) and the goal to attain closure, become positively related. That is, the higher NFC, the greater importance of scoring high on a task, as scoring high maximizes certainty that the task requirements will be met. Since in this case the importance of the task goal varies with NFC and the task goal requires effort, effort investment should be higher for high (vs. low) NFC individuals. In line with that Richter et al. (2012) found that participants high on NFC showed increased myocardial reactivity, interpreted as high effort investment, compared to participants low on NFC while performing a difficult categorization task. High (compared to low) NFC individuals were thus more motivated to invest effort to attain the task goal, when the task required effort.

However, when effortful and effortless means provide certainty that the task requirements will be met, task goal importance should be independent of NFC levels. It can thus have a separate, moderating, impact on the driving force, and thereby effort (CET). In support of that, Viola et al. (2015) found an interactive effect of NFC and outcome relevance (goal importance) on effort invested in a random dot motion task. High (vs. low) NFC individuals invested more effort in the task (had longer reaction times) when the outcome relevance was high as compared to when it was low.

Hence, we expect that in a situation wherein both effortful and effortless means are available and both are equally effective at providing closure, the relationship between NFC and effort would be moderated by task goal importance in a way that NFC would be related to lower effort but only when task goal is found unimportant. When the task goal is perceived as important, we do not predict a relationship between NFC and effort. In other words, high task goal importance should compensate for the tendency of high NFC individuals to select quick and easy means to closure.

## Means instrumentality

According to CET, the goal importance determines not only the willingness to invest effort but also the instrumentality of selected means to attain a goal. The greater the goal importance, the greater the likelihood to select most instrumental means, even if they pose considerable demands. So, when the task goal is to get as many points as possible and there is no easier way to meet the task requirements, investing a large amount of effort is the most instrumental strategy for the goal of closure, as it provides most certainty that the task requirements will be met (highest score will be earned). In this case, high NFC individuals would continue to follow this effortful strategy regardless of the information that alters the mindset to engage in less effortful means to solve the task. It is because high in NFC individuals should perceive effortful strategy as more instrumental for the focal goal. In support for this, Jaśko et al. (2015) have found that high (but not low) NFC individuals either increased or decreased their information search depending on which strategy (effortless or effortful) was perceived as more instrumental toward the goal of making an accurate decision.

To sum up, in line with CET we propose that when both more and less demanding means to achieve closure are available and both are equally instrumental for that goal (Study 1), NFC should be related to less effort invested in the task, unless the task goal is perceived as important. However, when closure may be achieved via demanding means only (Study 2), high NFC should be related to greater effort invested in the task and there should be no moderating effect of task goal importance. Furthermore, when there are both, more and less demanding means available, but the effortful one is more instrumental for the goal of closure (Study 3), high NFC individuals should select the most instrumental (and effortful) strategy to attain their goal.

## Overview of the studies

To test our predictions, we conducted three studies in which we asked participants to perform a task related to the goal of getting as many points as possible in a complex multiple task environment designed to maximize the influence of effort, rather than ability, on task performance. The entire task consists of 25 main tasks (i.e., tasks that are awarded points and thus contribute to the main task goal), which comprise various reasoning and logical puzzles, as well as other mundane tasks (i.e., not contributing to the main task goal but introduced to make the task more complex and engaging). Participants’ explicit aim was to get as many points as possible by completing selected main tasks. Study 1 incorporated the situation in which attaining closure may be quick and easy, or long and difficult. Participants had an option to “quit the task” after completing six tasks. In other words, they were instructed that they were required to get as many points as they could, however, if they thought they had achieved the goal, they had the opportunity to quit the task. Quitting early reflected a lack of effort investment. So, the task requirements were met when either of the two options was selected (as explicitly mentioned in the instructions) and both options were equally instrumental for the goal of closure. We predicted that NFC would be negatively related to effort investment, unless the task goal is perceived as being important. The differences in effort investment should translate into differences in performance, which is to say that increased effort investment should lead to better performance on the task.

In Study 2, attaining closure was possible only through effortful and laborious means. Since the easy-way out option was not provided and achieving closure could only be attained through effortful means, we expected a positive relationship between NFC and effort investment. Like in Study 1, effort investment should lead to better performance. We expected that task importance would not moderate this relationship, as regardless of perceived task importance, high NFC individuals should be more motivated to attain the task goal, i.e., get the highest score in the task (in this study, task goal importance and NFC should be positively related).

In Study 3, we tested a situation wherein there were also two strategies of performing the task available but one was more instrumental for the goal of closure than the other. To that aim, we used the same task version with no easy way out as in Study 2 and manipulated the suggestion of the optimal strategy to solve the task. Thus, in the effort enhancing condition, participants were told that the task goal is best attained by focusing mainly on logical thinking tasks which are more difficult and require more effort. In the effort minimizing strategy condition, participants were told that the task goal could be best attained by focusing mainly on the reasoning ability tasks, which are easier and require less effort.^1^ We predicted that high NFC individuals would invest a large amount of effort in task performance regardless of the suggested effort minimizing vs. enhancing strategy, as the effortful strategy provides more certainty that the task requirements would be met. On the other hand, we predicted that low NFC individuals would invest effort in line with our manipulation (more effort in the effort enhancing condition but less effort in the effort minimizing condition), as maximizing certainty does not drive their behavior. That is, we expected that low NFC individuals would adjust their effort level to perceived task difficulty (a pattern robustly found in effort studies, e.g., Richter et al. 2008; Wright 1996). By contrast, high NFC individuals would adjust their effort level to the task goal importance (Study 1) and select most instrumental, although not always the least effortful, strategies for the goal of closure (Study 3).

In all three studies a behavioral measure of effort investment was used (as in e.g., Jaśko et al. 2015; Roets et al. 2008; Viola et al. 2015). Specifically, we treated the time spent per task as an indicator of effort investment, assuming that the longer time a person spent on a given task, the greater his/her effort investment. A similar approach was incorporated by Viola et al. (2015), who indexed cognitive effort using reaction time (RT), on the assumption that higher RTs during a motion discrimination task represented a greater willingness to spend time viewing the motion display. Likewise, Freund (2006) used time spent per task as an indicator of the strength of motivation, or persistence in a goal pursuit. Also, in traditional motivational research, the persistence (i.e., time spent on a task) aspect of effort (Wright 2008) has been widely used as an operationalization of motivation (e.g., Deci 1971; Deci et al. 1999). This is based on the assumption that motivation energizes behavior and shields against competing goals or tendencies, so that the higher the motivation, the more persistence is seen amongst people working towards a goal.

## Study 1

In Study 1 we tested whether NFC predicted effort investment and thus performance in the cognitive task depending on perceived task goal importance. In this study, achieving closure was possible via both less demanding (quitting after completing minimal number of tasks) and more demanding (getting the highest score) means. Since getting the highest score was not necessary to achieve closure, we expected NFC and task goal importance to be uncorrelated and interactively predict effort invested in the task. We thus predicted that NFC would be negatively related to effort invested in the task, unless the task is perceived as important. Differences in effort should translate into differences in performance, i.e., there should be a negative effect of NFC on performance via effort but only when the task in perceived as unimportant.

## Method

### Participants

One hundred thirteen participants^2^ took part in the study (mTurk sample). All were awarded a monetary compensation of $3 for participation in the study. Data from three participants were deleted from the analysis as they spent less than 3 min on the task (time does not include reading the instructions) and most of the tasks were abandoned rather than completed. Thus, for further analyses, data from 110 participants were included (51 men, 59 women) aged between 19 and 67 years (*M* = 35.20, *SD* = 10.59).

### Measures

NFC was measured with a scale (Webster and Kruglanski 1994 in a version by Roets and Van Hiel 2011) consisting of 15 items, comprised of five subscales: Preference for Order, Preference for Predictability, Discomfort with Ambiguity, Closed-mindedness and Decisiveness. A sample item is “I don’t like situations that are uncertain.” Participants marked their responses on a scale from 1 (*completely disagree*) to 6 (*completely agree*). The overall score was calculated by averaging answers to all items. The higher the score, the higher the NFC (*Cronbach*’*s α* = 0.91; *M* = 4.49, *SD* = 0.89).

#### Experimental task

**Fig. 1:**
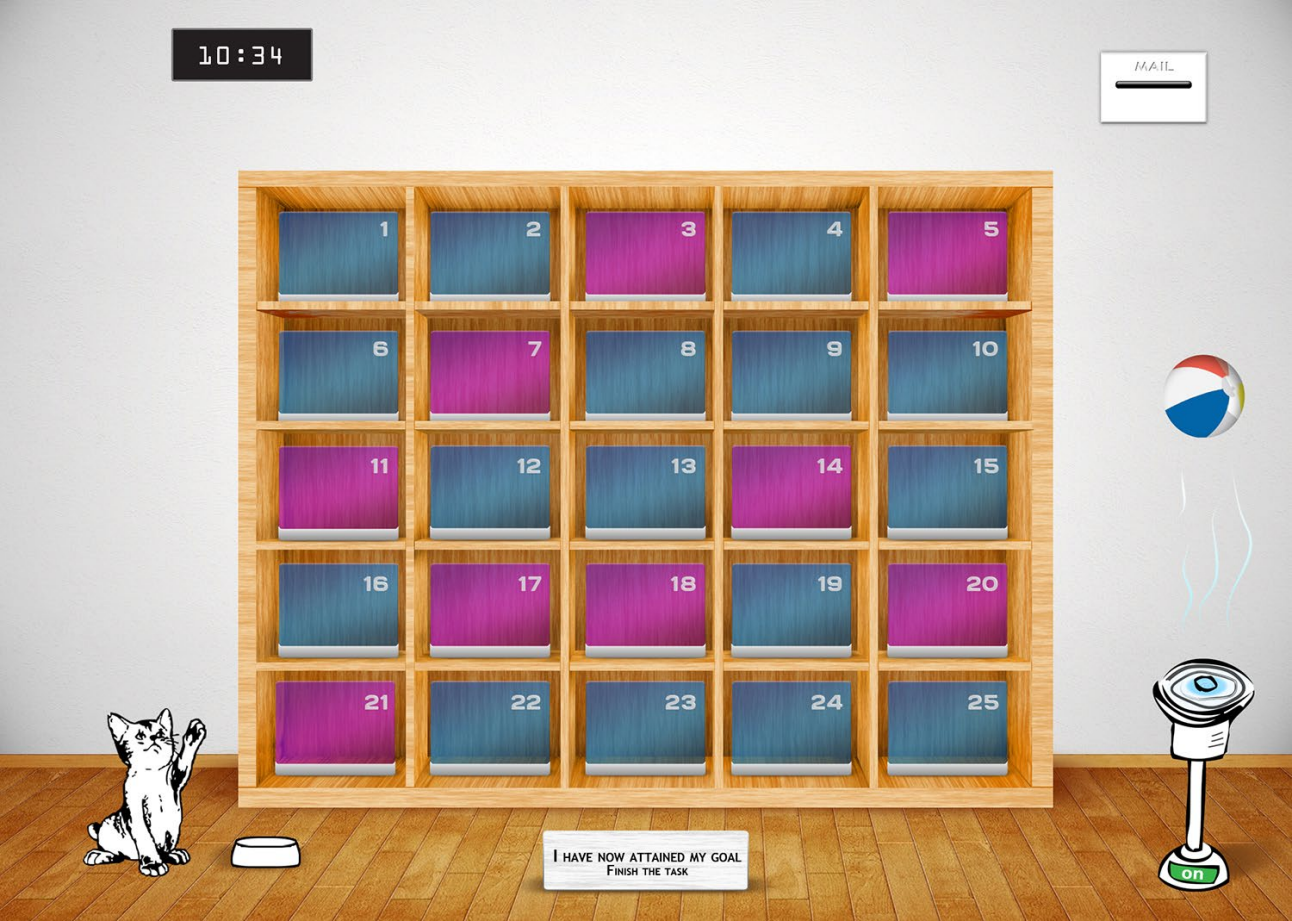
Sample screen from multiple task paradigm. Participants’ goal was to get as many points as possible by performing individual tasks. The tasks are selected by clicking on a given box, at which point a window with task-specific instructions appeared and the participant was presented with five items/questions of a given type (e.g., Raven’s matrices)

Participants were presented with a set of 25 tasks and were told that their aim was to get as many points as possible. The number of completed tasks and the order of their execution were up to the participants. The tasks were organized in a 5 × 5 matrix (see Fig. 1), with each box representing a task. There were two types of tasks: tasks named “logical” and “reasoning”. The former included items from Raven’s progressive matrices, number sequence and word analogies. The latter comprised category generation items, jumbled sentences and memory recognition items (see Supplementary Materials for task item examples). We wanted to use various types of tasks, so that performance on the entire task did not rely on one particular cognitive ability. Each task consisted of 5 items and participants were rewarded points in proportion to the number of items successfully completed. All participants were given two attempts per item to get the correct answer, so that they could perform well on the task if motivated to. There was no negative scoring for incorrect answers. Participants could quit each individual task at any point by clicking the “Quit” button presented in each task window.

Additionally, participants were instructed to perform some other, mundane tasks while performing the main tasks. Specifically, they were told to monitor a ball floating above the fan (when it dropped, one could correct its position by pressing a button on the fan); watch a mail box icon (when it started blinking, one could open it and read a “fun fact”); react to an alarm clock (when on, it could be turned off by a mouse click); and feed a hungry cat (when the cat was hungry it started meowing; it could be “fed” by clicking on the food bowl next to it). Mundane tasks were activated every 90 s in a random order. Mundane tasks were used to add complexity to the task situation and make it more attractive and engaging for participants.

There was a time limit of 45 min (pretested for most participants to complete all tasks). In order to introduce the idea of two routes to attain closure, a button titled “I have now attained my goal” would be activated after attempting six tasks. Participants could press it at any moment once it was activated. Thus, the task ended once (1) all 25 tasks were attempted, (2) if participants ran out of time or (3) they clicked the “I have now attained my goal” button.

Task performance was measured by simply calculating the total number of points earned from completing each of the main tasks (as that was the explicit task goal emphasized in the instructions). The points were calculated based on accuracy, as participants were awarded points only for items correctly performed. Effort was measured as a mean time spent per each task.

#### Task importance

Task importance was measured with an item presented after the study (*How important was it for you to attain the task goal?*; 5-point Likert scale anchored 1—*definitely not important* and 5—*definitely important* was used). The mean task importance score was *M* = 3.94, *SD* = 0.92.

### Procedure

The entire study was conducted in one session. Participants first completed demographic questions and the need for cognitive closure scale. After a 10-min break, they were presented with the experimental task (for which they were instructed to wear headphones). Then they assessed the task goal importance. On completing the task and rating the task goal importance, participants were debriefed and thanked.

## Results and discussion

Descriptive statistics for effort (i.e., the average time spent on tasks) and task performance (i.e., the total number of points earned) are presented in Table 1. As expected, NFC and task goal importance were not correlated, *r* = .08, *p* = .39.

**Table 1:** Descriptive statistics for effort and goal attainment measures in Study 1 (N = 110) and Study 2 (N = 89)

	Study 1		Study 2	
	M	SD	M	*SD*
Total points	41.04	26.97	77.01	18.44
Tasks completed^a^	8.52	5.19	21.26	3.56
Time per task[s]	78.24	40.75	76.16	30.06

^a^Tasks completed are the ones in which a participant attempted and answered all five items/questions

**Fig. 2:**
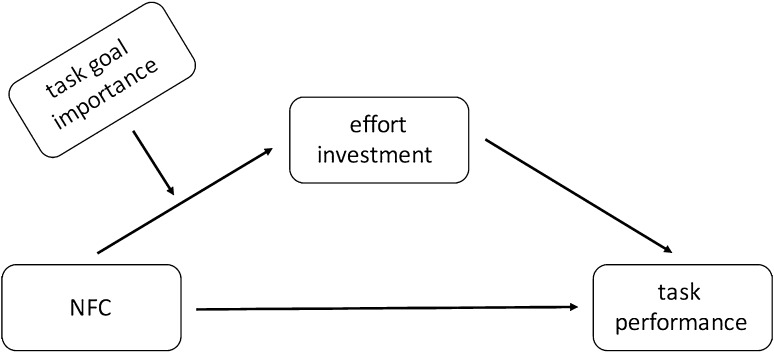
Theoretical model of the relationship between NFC, task goal importance, effort investment and task performance (Study 1)

To test our predictions, we checked whether NFC is associated with effort investment in the task (mediator), which in turn helps performance (DV). Additionally, we predicted that NFC was negatively related to effort and thus performance when individuals find the task goal unimportant, as compared to the situation when they find the task goal important (moderator). The model we tested is depicted in Fig. 2 (model 7 in Process macro for SPSS, Hayes 2013). To probe significant interactions, we used a simple slope analysis and calculated the effect of our IV on the DV at low (−1 SD) and high (+1 SD) values of the moderator. NFC and goal importance scores were mean-centered prior to analyses. To test moderated mediation effects, we utilized an index of moderated mediation (Hayes 2013). In each case 10,000 bootstrap samples were used, 95% bias corrected confidence intervals are reported. Due to a wide age range, age was controlled for in the analysis.

**Fig. 3:**
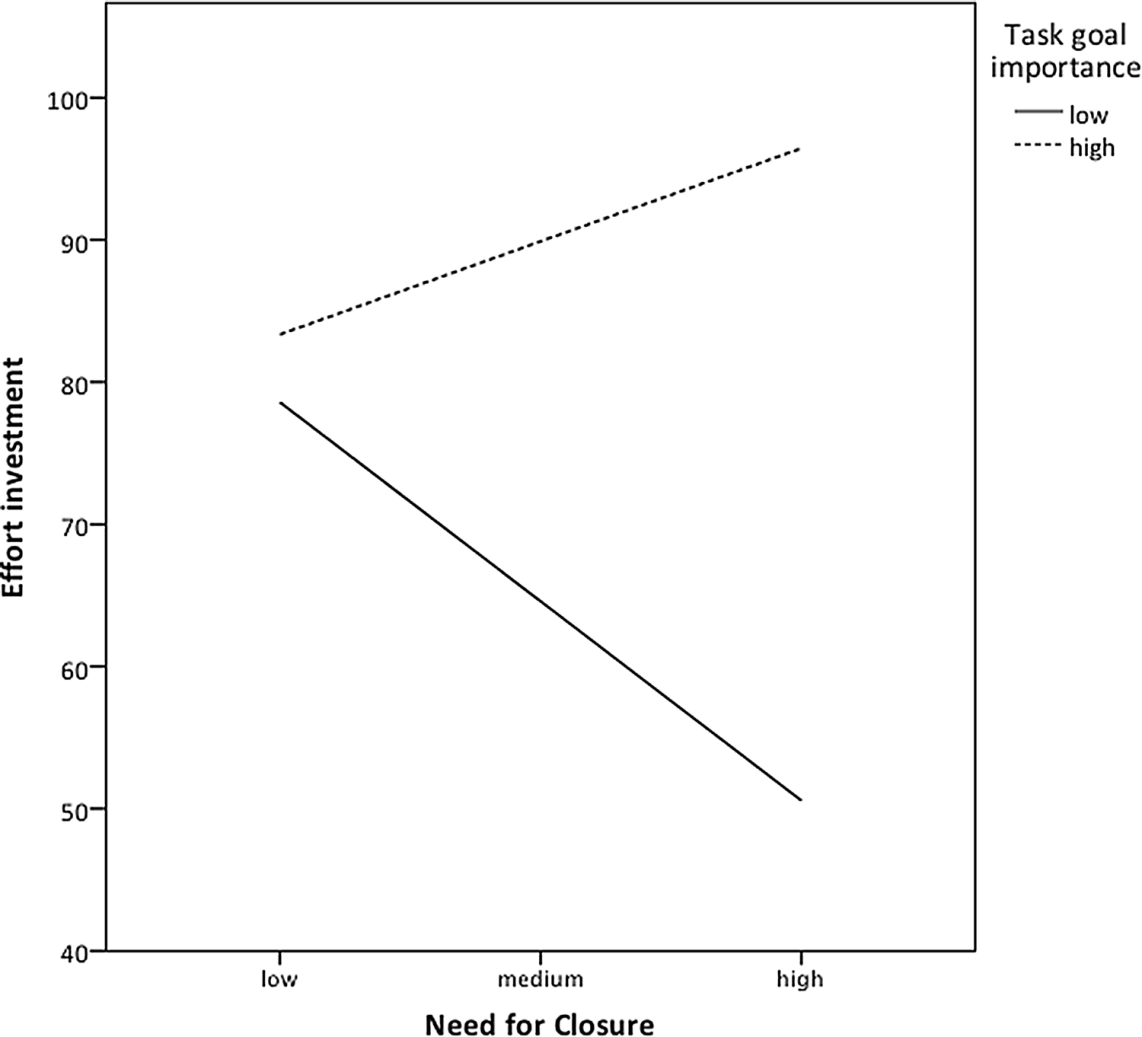
Relationship between NFC, task goal importance and effort investment (Study 1)

The results revealed the significant interactive effect of NFC and task goal importance on effort investment, *b* = 13.17, *SE* = 5.52, *t* = 2.38, *p* = .02, 95% *CI* [2.21, 24.12]. As predicted, the effect of NFC was significant only for low, *b* = −15.88, *SE* = 7.35, *t* = −2.16, *p* = .03, 95% *CI* [−30.46, −1.30], but not high, *b* = 7.18, *SE* = 5.59, *t* = 1.28, *p* = .20, 95% *CI* [−3.91, 18.27], task goal importance. The effect of NFC was negative but non-significant, *b* = −4.35, *SE* = 4.33, *t* = 1.01, *p* = .32, 95% *CI* [−12.93, 4.23], and the effect of task goal importance was significant, *b* = 14.53, *SE* = 4.31, *t* = 3.37, *p* = .001, 95% *CI* [5.98, 23.07]. The interaction is graphically presented in Fig. 3.

Furthermore, effort significantly predicted task performance, *b* = 0.18, *SE* = 0.06, *t* = 2.84, *p* = .006, 95% *CI* [0.05, 0.30], and there was a significant indirect effect of NFC through effort investment on task performance moderated by task goal importance (index of moderated mediation equal to *IMM* = 2.31, *SE* = 1.22, 95% *CI* [0.41, 5.37]). As predicted, there was a negative effect of NFC on performance via effort investment, but only when perceived task importance was low, *IE* = −2.79, *SE* = 1.32, 95% *CI* [−5.99, −0.72]. When perceived task goal importance was high, the effect was non-significant, *IE* = 1.26, *SE* = 1.34, 95% *CI* [−1.03, 4.41]. The direct effect of NFC on task performance was non-significant when effort investment was included in the model, *DE* = −5.00, *SE* = 2.79, *t* = −1.79, *p* = .08, 95% CI [−10.54, 0.54].

Additional inspection of differences between high and low task goal importance revealed that there were no significant differences for the effect of task goal importance on effort for low NFC individuals (*b* = 2.83, *SE* = 5.89, *t* = 0.48, *p* = .63, 95% *CI* [−8.85, 14.51]) but there were significant differences for high NFC individuals (*b* = 26.22, *SE* = 7.20, *t* = 3.64, *p* < .001, 95% *CI* [11.96, 40.49]).

The results thus revealed that NFC was negatively related to effort, and thus performance, for low task goal importance. For high task goal importance, this relationship was non-significant. When high NFC individuals perceived the task to be unimportant, they spent less time on individual tasks, which resulted in lower performance. In contrast, when the overall task was perceived to be important, the negative effect was no longer significant, i.e., task importance compensated for the tendency of high NFC individuals to select quick and easy routes to closure when they are available. These findings are in line with previous studies showing effort reducing tendencies amongst high NFC individuals (Kruglanski and Webster 1996), but add significantly to them by showing that when the task goal is seen as important, effort is not reduced. In line with CET (Kruglanski et al. 2012), we suggest that for high NFC individuals who found the task important, the driving force (perceived task importance) matched the restraining force (task difficulty) and therefore they engaged in efficient effort investment. When high NFC individuals did not perceive the task to be important, they did not invest effort in the task. In other words, they found the “easy way out” to attain closure. Low NFC individuals, on the other hand, regardless of the level of task goal importance did not differ in how much effort they invested. Hence, only high NFC individuals adjusted their effort level in response to their current task goals, whereas the motivation levels of low NFC individuals were mostly unaltered.

## Study 2

Study 1 demonstrated that NFC was associated with decreased effort investment, which led to worse task performance, unless the task was perceived as important. It is important to note that in Study 1 the task could be terminated any time. This is a comfortable situation for high NFC individuals as it affords an easy, yet efficient, option for closure. In Study 2, we investigated whether NFC was associated with increased effort investment, and thus better task performance, in a task that can be completed only via demanding means, i.e., via working towards the highest score in the task. Since the latter is the only way to gain certainty that the task requirements will be met, we expected that it would be more important for high than for low NFC individuals to perform well on the task (NFC and task goal importance should be positively correlated). Therefore, we predict that NFC would be related to greater effort invested in the task, which should translate in better performance, and this effect should not be moderated by task goal importance.

### Participants

The sample comprised one hundred and four^3^ mTurk users (55 women, 49 men) aged between 21 and 57 years. Due to a coding error, only partial data for first 15 participants were saved. Therefore, these cases were not included in further analysis. The final sample comprised 89 (43 men, 46 women) participants. The age ranged from 21 to 49 years with the mean of *M* = 33.18 (*SD* = 7.87). Participants were given a monetary compensation of $3 for participation in the study.

### Measures

All measures used were the same as in Study 1, except that there was no “I have now attained my goal” button incorporated in the multiple task paradigm. Thus, the task ended once all 25 tasks were attempted or the 45-min time window had elapsed.

### Procedure

As in Study 1, participants were first asked to complete demographic questions and the need for cognitive closure scale (α = 0.92, *M* = 4.08, *SD* = 1.04). Then they were presented with the experimental task, the goal of which was to get as many points as possible. As in Study 1, participants were subsequently asked to assess task goal importance (*M* = 4.02, *SD* = 0.83). In the end, they were debriefed and thanked.

## Results and discussion

Descriptive statistics for performance are presented in Table 1. Unlike in Study 1, NFC and task goal importance were significantly positively correlated, *r* = .32, *p* = .002.

**Fig. 4:**
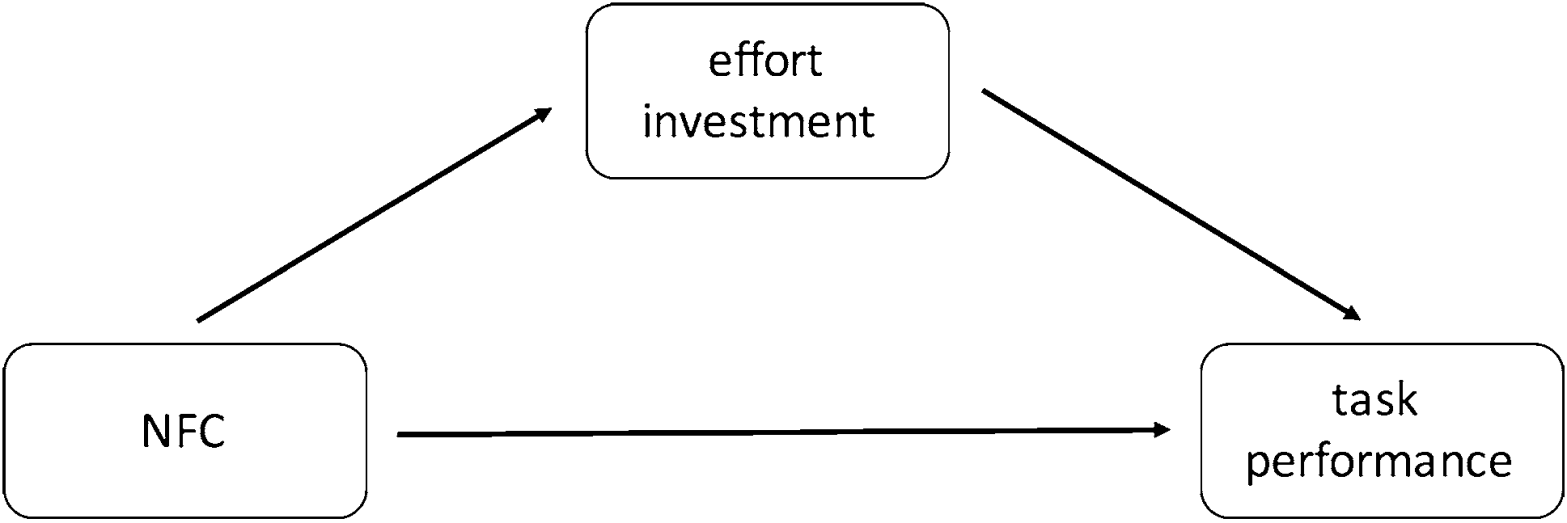
Theoretical model of the relationship between NFC, effort investment and task performance (Study 2)

To test the effects of NFC and effort investment on task performance, we ran a mediation analysis with the use the Process macro for SPSS (Hayes 2013; model 4, see Fig. 4). Since in this study the task ended when either all tasks were completed or participants ran out of time, differences in time spent per task could be attributable to the differing number of tasks completed (the more tasks completed within the fixed time, the less time per task). So, in the analyses we controlled for the number of completed tasks. Thus, the obtained effects can be interpreted as differences in effort investment for those who completed the same number of tasks.

The results showed that NFC significantly predicted effort (time spent per task), *b* = 7.70, *SE* = 3.84, *t* = 2.01, *p* = .048, 95% *CI* [0.06, 15.34], and effort significantly predicted task performance (the number of points earned), *b* = 0.14, *SE* = 0.04, *t* = 3.59, *p* < .001, 95% *CI* [0.06, 0.22]. Using 10,000 bootstrap samples, we found an indirect effect of NFC on task performance through effort investment, *IE* = 1.11, SE = 0.66, 95% *CI* [0.13, 2.79]. The relationship between NFC and task performance remained non-significant when effort investment was entered into the regression model, *DE* = 2.26, *SE* = 1.47, 95% *CI* [−0.66, 5.18]. The total effect of NFC was positive and significant, *TE* = 3.37, *SE* = 1.53, *t* = 2.20, *p* = .03, 95% *CI* [0.33, 6.42]. Thus, the higher NFC, the more time spent per task, which translates into more points earned in the overall task. The relationship was not dependent on task goal importance (neither the NFC × goal importance interaction significantly predicted effort invested in the task, *b* = −0.29, *SE* = 3.85, *t* = −0.08, *p* = .94, 95% *CI* [−7.96, 7.37], nor there was a significant moderated mediation index for model 7, *IMM* = −0.04, *SE* = 0.56, 95% *CI* [−1.09, 1.31]). Moreover, in this study, unlike in Study 1, NFC significantly predicted goal importance, goal importance predicted effort, which in turn predicted task performance (a significant double mediation, *b* = 0.82, *SE* = 0.43, 95% *CI* [0.24, 2.12]; see Supplementary Materials for testing the double mediation hypothesis for all three studies^4^).

The results supported our hypotheses, as there was a positive indirect effect of NFC on task performance mediated by effort invested in the task. Thus, in this study, opposed to the typical findings on NFC (see Kruglanski 2004, for an overview) and the general tendency of high (vs. low) NFC individuals to conserve resources (Kruglanski et al. 2012), NFC was positively related to effort invested in a cognitive task. This is in line with other studies showing the “ironic effects” of NFC (e.g., Klein and Webster 2000; Kruglanski et al. 1991, 1993).

As expected, this relationship was not moderated by task goal importance, as closure might be achieved only by using effortful means. In other words, as only by investing effort were high NFC individuals able to achieve their goal (i.e., closure), they adjusted their effort level to that goal and the current task requirements. Moreover, in this situation, unlike in Study 1, NFC was positively related to task goal importance. We argued that because best task performance is the only way to attain closure, its importance increases for high (but not low) NFC individuals. This increased importance explained heightened effort and better task performance (as suggested by the significant double mediation).

## Study 3

In this study, we checked whether NFC was related to selection of the most instrumental strategy to attain closure. When the task goal is to get as many points as possible and there is no easier way to meet the task requirement, investing a large amount of effort is the most instrumental strategy for the goal of closure, as it provides most certainty that the task requirements will be met (highest score will be earned). We wanted to verify whether high NFC individuals would continue to follow this strategy regardless of the information that alters the mindset to engage in less effortful means to solve the task. To manipulate the mindset, we provided participants with an information suggesting that investing effort (in the effort enhancing condition) or minimizing effort (the effort minimizing condition) was the optimal way to perform the task. We thus presented participants with two strategies to perform the task, that would either prompt them to maximize or minimize their effort. Importantly, the former strategy was more instrumental for closure than the latter, as it led to a high score more certainly. We then tested whether NFC predicted effort investment and task performance in each of the two conditions.

We expected that high NFC levels would be related to high levels of effort in both, effort enhancing and effort minimizing, conditions. Low NFC levels, on the other hand, should be related to high effort in the effort enhancing, but not in the effort minimizing condition. That is, low NFC individuals would not be motivated to enhance their effort when suggested otherwise, as they are not motivated by striving for maximizing certainty. In other words, NFC should be positively related to effort investment in the effort minimizing condition but there would be no significant relationship in the effort enhancing condition as in this condition all participants would invest large amount of effort. We again do not expect a moderating effect of task goal importance as we assume that task would be more important for high (than low) NFC individuals in a situation when getting the most points is the best way of achieving closure. Differences in effort should translate into differences in performance, i.e. NFC should significantly predict performance in the effort minimizing condition (in the effort enhancing condition, performance in the task should be high for both low and high NFC individuals).

### Participants

The sample comprised 88 participants (*N* = 88, 37 women, 51 men) aged between 20 and 50 (*M* = 33.22, *SD* = 8.18). The study was conducted via mTurk. All participants were given a $3 monetary compensation for the participation in the study.

### Measures

All measures used were the same as in Study 2 except for the suggestion of the optimal strategy to solve the task, which was incorporated into the experimental task. The suggested effort investment strategy was manipulated via information that was presented at end of the task instructions. Participants were randomly assigned to one of two conditions. In the effort enhancing condition they were informed about prior research in which it was found that focusing on the logical tasks was the best strategy to get the most points, because while the tasks are difficult and require more time and effort, they are worth more points. In the effort minimizing condition, participants read about previous research reporting that the most effective strategy was to focus on the reasoning tasks, as these tasks are easy and participants may correctly solve more of them (than the difficult, logical tasks). In both conditions, participants were required to solve all task and the manipulation was aimed at drawing their attention to either difficult or easy tasks, which should motivate them to either maximize or minimize their effort (Ach 1935; Brehm and Self 1989; Kruglanski et al. 2012; Wright 1996, 2008).

### Procedure

As in previous studies, participants were first asked to complete demographic questions and the need for cognitive closure scale (the scale proved a satisfactory reliability of *Cronbach’s α* = 0.85). Then they were presented with the experimental task, the goal of which was to get as many points as possible. Each participant was randomly assigned to one of the two conditions in which either an effort enhancing or an effort minimizing strategy was suggested. As in Study 2, the task ended once all 25 tasks were attempted or the 45-min time window has elapsed. Also, as in Study 1 and 2, at the end of the task the participants were asked to assess the importance of the task goal. They were then debriefed and thanked.

## Results and discussion

### Manipulation check and preliminary analysis

The results show that on average participants spent more time per task in the effort enhancing (*M* = 95.28 s, *SD* = 37.16) than the effort minimizing (*M* = 78.46 s, *SD* = 35.82) condition, *F* (1, 86) = 4.67, *p* = .03. They also tended to score more points in the effort enhancing (*M* = 93.74, *SD* = 23.37) than the effort minimizing (*M* = 83.76, *SD* = 27.07) condition, *F* (1, 86) = 3.42, *p* = .07. Thus, our results show that the manipulation was effective and that more effort was exerted in the effort enhancing than the effort minimizing condition.

The mean NFC score was *M* = 4.09 (*SD* = 0.70), the mean task goal importance was *M* = 4.05 (*SD* = 0.93), and the two measures were positively correlated, *r* = .22, *p* = .04.

### Hypothesis testing

**Fig. 5:**
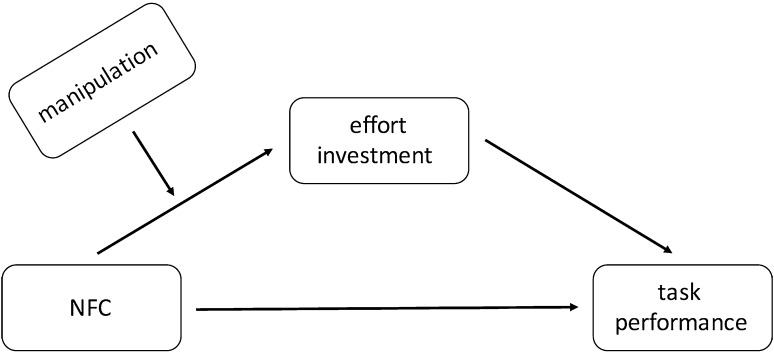
Theoretical model of the relationship between NFC, effort investment, task performance and effort enhancing versus minimizing condition (Study 3)

Similar to previous studies, we tested whether NFC was associated with task performance (the number of points earned; DV) via effort investment (time on task; mediator). In addition, we tested whether the manipulation (effort enhancing vs. minimizing condition) played a moderating role. The model we tested is pictured in Fig. 5 (model 7 in Process macro for SPSS, Hayes 2013). To probe significant interactions, we used a simple slope analysis and calculated the effect for each of the two conditions. To test moderated mediation effects, we utilized an index of moderated mediation (Hayes 2013). In each case 10,000 bootstrap samples were used, and 95% bias corrected confidence intervals were reported. The effort enhancing strategy condition was coded as 1 and the effort minimizing strategy condition as 0. We controlled for the number of tasks completed.

**Fig. 6:**
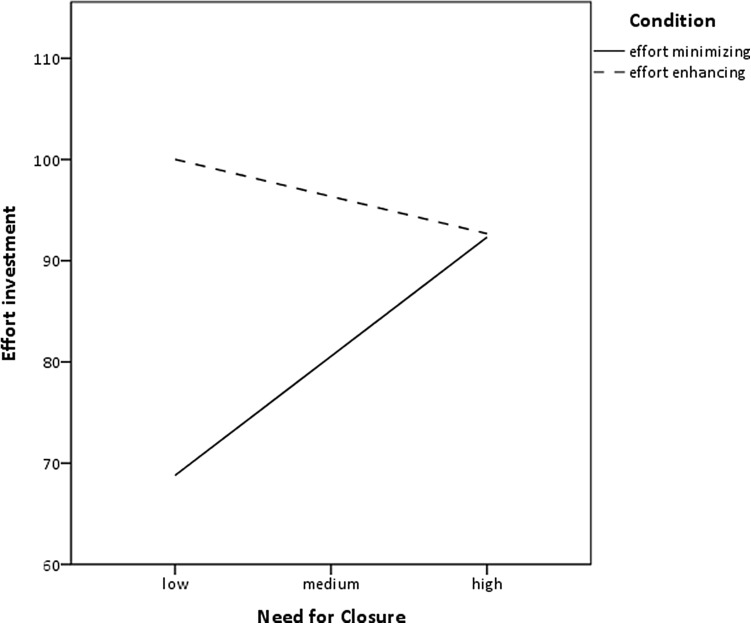
Relationship between NFC, effort enhancing versus minimizing condition, effort investment, and task performance

In line with our predictions, we found a significant interactive effect of NFC and condition on effort, *b* = −22.21, SE = 10.20, *t* = −2.18, *p* = .03; 95% *CI* [−42.50; −1.93]. The effect of NFC was also significant, *b* = 16.93, *SE* = 7.18, *t* = 2.36, *p* = .02; 95% *CI* [2.65; 31.21]; as was the effect of condition, *b* = 106.57, SE = 42.32, *t* = 2.52, *p* = .01; 95% *CI* [22.41; 190.74]. Simple slope analysis revealed that NFC was positively related to effort investment among individuals in the effort minimizing condition (*b* = 16.93, *SE* = 7.18, *t* = 2.36, *p* = .02; 95% *CI* [2.65; 31.20]) but the effect was non-significant in effort enhancing condition (*b* = −5.28, *SE* = 7.21, *t* = −0.73, *p* = .47, 95% *CI* [−19.62, 9.06]). In addition, inspection of differences between the two conditions revealed that low NFC individuals invested significantly more effort in the effort enhancing condition compared to the effort minimizing condition (*b* = 31.22, *SE* = 9.99, *t* = 3.13, *p* = .002, 95% *CI* [11.36, 51.09]), whereas high NFC individuals did not differ in their effort investments between the two conditions (*b* = 0.35, *SE* = 9.96, *t* = 0.04, *p* = .97, 95% *CI* [−19.46, 20.16]). The interaction is graphically presented in Fig. 6.

Moreover, effort significantly predicted task performance, *b* = 0.12, *SE* = 0.04, *t* = 3.45, *p* < .001, 95% *CI* [0.05, 0.20] and the whole moderated mediation model was significant (*IMM* = −2.77; *SE* = 1.61, 95% CI [−6.60, −0.41]). NFC was positively related to task performance through effort investment. However, this relationship was significant among participants in the effort minimizing condition (*IE* = 2.11, *SE* = 1.24, 95% *CI* [0.32; 5.17]), but not among those in the effort enhancing condition (*IE* = −0.66, *SE* = 0.79, 95% *CI* [−2.48, 0.71]). The direct effect of NFC on task performance was non-significant, *DE* = 0.24, *SE* = 1.75, 95% CI [−3.25, 3.72].

Furthermore, as expected task goal importance was not a significant moderator of this relationship (*b* = −10.75, *SE* = 13.77, *t* = −0.78, *p* = .44 for the three-way interaction). Also, as in Study 2, NFC was positively related to goal importance, which via effort predicted better task performance (significant double mediation, *IE* = 0.28, *SE* = 0.23, 95% *CI* [0.02, 1.00], see Supplementary Materials for more details).

Study 3 was conducted to test the situation in which there are two strategies to closure, effortful and effortless, but one is more effective than the other (one provides more certainty that the task requirements will be met). The strategy was manipulated by introducing the cue that informed participants which means (effort enhancing or effort minimizing) are optimal for the task at hand. We demonstrated that high NFC was related to heightened effort investment, and thereby high task performance, regardless of the manipulation. By contrast, low NFC was related to more effort in the effort enhancing condition and less effort in the effort minimizing condition thus following the classic difficulty trend in effort studies (e.g. Brehm and Self 1989; Wright and Brehm 1984). In other words, participants low in NFC adapted to the situational demands and performed according to the information provided. In contrast, high NFC individuals adapted their efforts to their salient goal, namely, the goal of attaining closure. In the effort enhancing condition, both high and low NFC individuals were equally motivated to invest effort, however, the difference was seen in the effort minimizing condition, wherein participants low in NFC followed the mindset to invest less effort, and therefore got fewer points, while people high in NFC still engaged in heightened effort. Moreover, mimicking the results of Study 2, Study 3 found that, as long as the task requires the best score and closure is best attained by the demanding means, the task goal importance does not moderate the relationship between NFC and effort. In fact, task goal importance was positively related to NFC and mediated the relationship between NFC and effort. This is in line with our reasoning that in case wherein best task performance (effortful means) in the most instrumental strategy to achieve closure, the importance of that means increases for high, but not low, NFC individuals.

## General discussion

An abundance of research has demonstrated that NFC is linked to decreased effort investment in judgments and decision-making and showed a preference for effortless cognitive strategies (Kruglanski 2004, for overview). Some studies, however, have showed that NFC might also lead to effortful cognitive strategies under certain conditions (e.g., Klein and Webster 2000; Kruglanski et al. 1991, 1993; see; Roets et al. 2015, for overview). Our studies add to these findings by demonstrating that NFC can lead to both effortless and effortful strategies adapted in cognitive tasks depending on whether closure can be attained via more or less demanding means in a given situation. Specifically, when both means to closure are available and both are equally instrumental to the goal of closure, NFC is related to less effort invested in the task, unless the task is perceived as important. This finding is in line with the typical “cognitive miser” effects of NFC (Kruglanski and Webster 1996) and with the notion that NFC might be related to a tendency to conserve cognitive resources (Kruglanski et al. 2012). However, it also shows that if the task goal is perceived to be important, effort investment is not reduced (and might even increase) among high NFC individuals. Our results are therefore in line with CET (Kruglanski et al. 2012), which assumes that the higher the task goal importance, the more motivated a person is to exert effort and the more likely s/he is to choose more demanding means to attain that goal (see also Atkinson and Birch 1970).

However, when it is possible to attain closure only via more demanding means, high NFC is associated with increased effort investment, independent of the perceived importance of the task (Study 2). This effect held among high NFC individuals, regardless of the manipulation of the suggested ‘best strategy’ for goal attainment (effort enhancing vs. effort minimizing, Study 3). Low NFC individuals invested more effort in the effort enhancing condition, but less effort in the effort minimizing condition. This shows that when there are two strategies to closure available but one is less instrumental to the goal of closure than the other, high NFC is related to selection of the more instrumental one, even if it requires effort. This is in line with CET, which postulates that the higher the potential driving force (goal importance), the greater the probability that one will choose more demanding means to attain the goal. Selecting most instrumental means, as well as adjusting the effort level to the task goal importance, shows that high NFC individuals can flexibly adjust their processing strategy to most efficiently satisfy their salient goals and inner states.

It should be noted, however, that in our study the selection between more and less demanding means was operationalized at the level of the entire task engagement rather than at the level of individual tasks. So, we analyzed whether participants invested high versus invested low effort in the task rather than whether they selected logical (7-point) versus reasoning (3-point) tasks. However, differences at the level of individual tasks (selecting 7- vs. 3-point tasks) could emerge if the time limit was stricter and participants had to prioritize tasks. If that was the case, one might expect that high NFC individuals would be more likely to select more instrumental (7-point) tasks than low NFC individuals, but only when the easy way to attain closure is made unavailable or when they perceive the task to be important. This argument would be in line with Viola et al.’s (2015) study showing that high NFC individuals invested more effort in the task when the outcome relevance was high (as compared to when it was low). This, however, calls for experimental verification.

Our results show that NFC is related to lower effort when there are two efficient ways to closure available (via investing effort or using the “easy way out” option, Study 1). This does not imply, however, that high NFC individuals will usually use the easy way out when it is available. First, it depends on the importance of the task goal, as shown by the significant interaction obtained in Study 1. Second, both ways to closure in this study (unlike in Study 3) were equally instrumental to the goal of closure and permissible by the task instructions. Participants were told they could quit the entire task after completing a minimum of six individual tasks. If that was not the case and there was a “shortcut” that participants could use but it would not be in line with task rules, high NFC individuals might not be more prone to use it. Previous studies have shown that high NFC individuals are more motivated to follow situational norms and rules (Fu et al. 2007; Jaśko et al. 2015; Kruglanski and Webster 1996) and to comply with task demands (Jia et al. 2014) than low NFC individuals. Therefore, different results might be obtained depending upon how the various routes to closure relate to task requirements and task rules. Besides, the results of Study 3 show that it is not the effort minimizing tendency that guides the behavior of high NFC individuals but rather satisfaction of the goal of closure. If the former was the case, high NFC individuals should invest less effort in effort minimizing and more effort in effort maximizing condition. Our results thus support the recent notion that this not the means (effortful vs. effortless processing strategies) but the goal (attaining closure) that are crucial to a proper understanding of NFC (Jaśko et al. 2015; Kossowska et al. 2016; Roets 2016; Szumowska and Kossowska 2017).

The studies we described have a few limitations. First, many effort theories emphasize the role of resources or (perceived) ability in determining the amount of effort exerted in a task (Kruglanski et al. 2012; Brehm and Self 1989; Wright 1996, 2008; Wright and Brehm 1984). We have not measured resources or ability in our study, as we focused on NFC related differences in effort investment. To that aim, we made sure the tasks we used were not too difficult and pre-tested them before running the actual experiments. What is more, a 45-min timeframe was provided to solve the task (a timeframe in which most participants finished all of the presented tasks). Additionally, in each case participants were given another attempt to correct their answer and the tasks we used were diversified (there were logical and number sequence puzzles, memory tasks, category generation items etc.), so that success in the overall task was not based on one specific cognitive ability. Our results also show that performance in the task, as well as the effort indicator (the time spent per task), were significantly influenced by the mindset manipulation we used in Study 3. Thus, we can say that the differences we captured were motivational rather than ability-related. Including resources, however, would be advisable in future studies.

Another limitation is the effort index we used. Like other researchers (e.g. Deci 1971; Deci et al. 1999; Freund 2006; Viola et al. 2015), we used time spent per task as a behavioral measure of effort, whereby we assumed that the longer one spends on a given task, the more motivated s/he is to perform it well. However, we are aware that time per task might also be affected by participants’ ability to perform a certain type of tasks. Although we used different types of tasks, so that the results would not depend on one kind of ability, participants’ resources might have some influence on the time they spent working on a given task and longer times might be indicative of lower ability. This, however, does not seem to be the case in our studies, as in all of them the mean time per task positively predicted total points awarded, thus suggesting that it was not inability to correctly perform the task that was responsible for differences in the time spent on each task. So, even though the measure we used in our studies is not perfect, we decided that it would serve better than a self-report measure (see Schwerdtfeger 2004; Wilson and Dunn 2004) or than performance itself. It would be useful, however, to test whether similar results would be obtained if other (e.g. psychophysiological, Richter et al. 2012; Roets et al. 2008; Wright 1996, 2008) measures of effort were used. Also, it would be useful to implement an effort measure which would allow to differentiate between effort invested at a point in time and effort distributed across time (distinction we are here unable to make).

The results we obtained show that high levels of NFC might lead to both effortless and effortful strategies adapted in cognitive tasks depending on whether closure can be attained via more or less demanding means and depending on the importance of the task goal. These insights make a meaningful contribution to the NFC theory and to our understanding of the role played by effort investment in social cognition.

## Electronic supplementary material

Below is the link to the electronic supplementary material.

Supplementary material 1 (PDF 7663 KB)

## Footnotes

1 In this study participants were required to complete all tasks, regardless of the condition, so we assumed that drawing their attention to either type of tasks would affect their motivation level. Many motivational theories state that perception of task difficulty affects effort invested in the task, i.e., effort is not exerted in easy tasks (Ach 1935; Brehm and Self 1989; Kruglanski et al. 2012; Wright 1996, 2008), however demanding tasks motivate an individual to invest effort (see also Locke and Latham 2006).

2 We have run an a priori power analysis (G*Power 3.1, Faul et al. 2009) with a medium effect size of *f* ^*2*^ = 0.15 and power at 0.80 (Cohen 1988). It showed that a sample of at least 55 participants would be necessary for a multiple regression model (R^2^ increase due to adding a single regression coefficient) to obtain the assumed power. However, we were interested in estimating the whole moderated mediation model rather than single coefficients. Therefore, we have decided to recruit more participants than suggested by the results of the analysis (the research on the sample size selection of power derivation for the index of moderated mediation is still emerging). We did not conduct any statistical analyses before we finished collecting the data. The same was the case for Studies 2 and 3.

3 A similar study was run on a smaller sample size (*N* = 46) and the same results were obtained (see Supplementary Materials for the description of results).

4 These analyses are presented in Supplementary Materials because the double mediation hypothesis was not the main one tested in our studies and supports our reasoning only indirectly.
