# Supplementary material for: “When the going gets tough, the tough get going”: Motivation towards closure and effort investment in the performance of cognitive tasks
Source: Motiv Emot. 2017 Apr 19;41(3):308–21. doi: 10.1007/s11031-017-9613-y (PMC5442255; doi:10.1007/s11031-017-9613-y)
Supplement: Supplementary file 1 — Supplementary material 1 (PDF 7663 KB) [file 11031_2017_9613_MOESM1_ESM.pdf]

TASK EXAMPLES

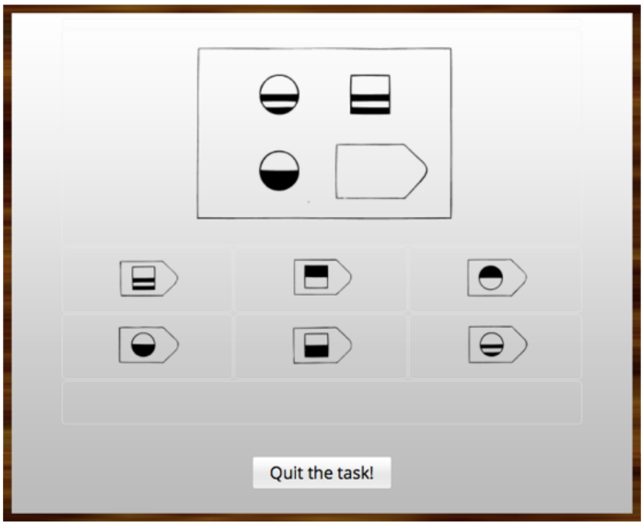

Figure 1. Raven's matrix
Participant's task is to identify the missing element that completes the pattern by selecting one of the presented options.

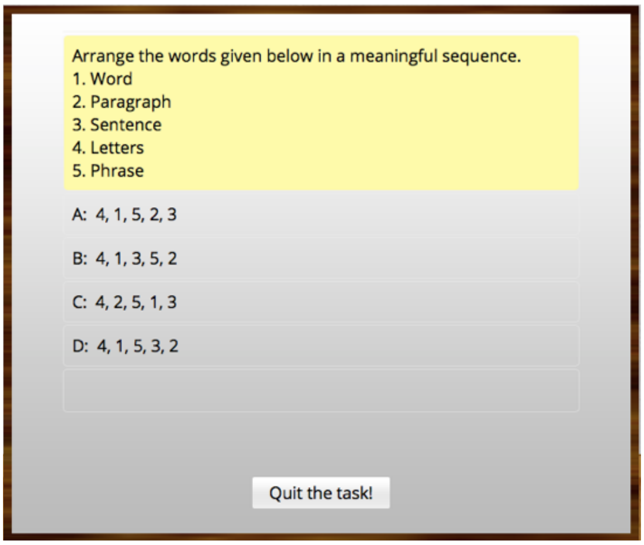

Figure 2. Logical sequence
Participant's task is to select the right sequence by clicking on one of the presented options.

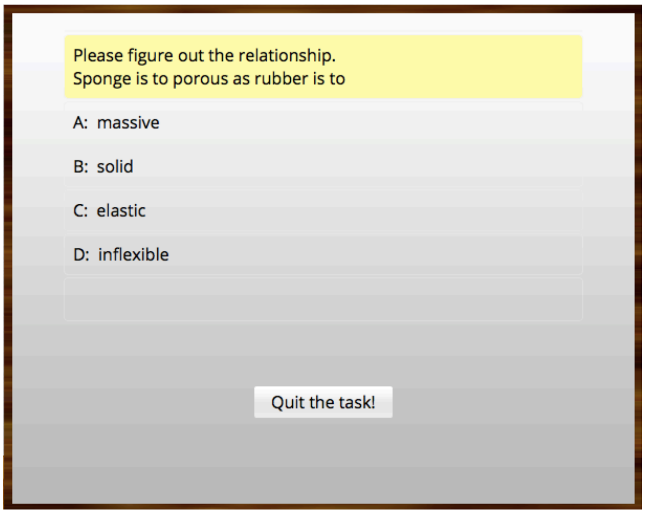

Figure 3. Word analogy
Participant's task is to select the right word by clicking on one of the presented options.

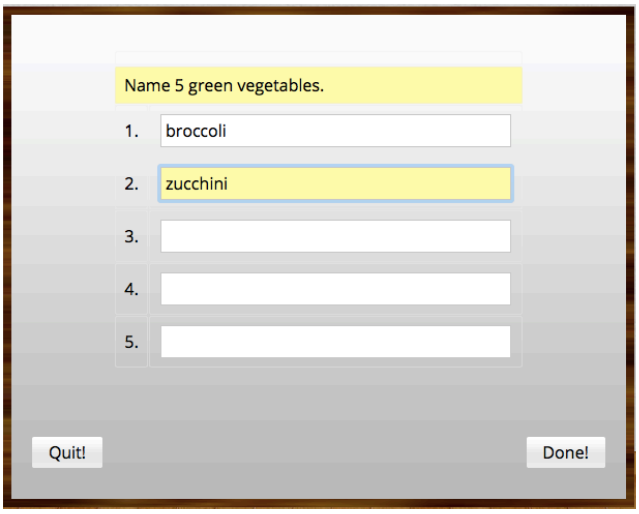

Figure 4. Category generation
Participant's task is to list out words for a given category.

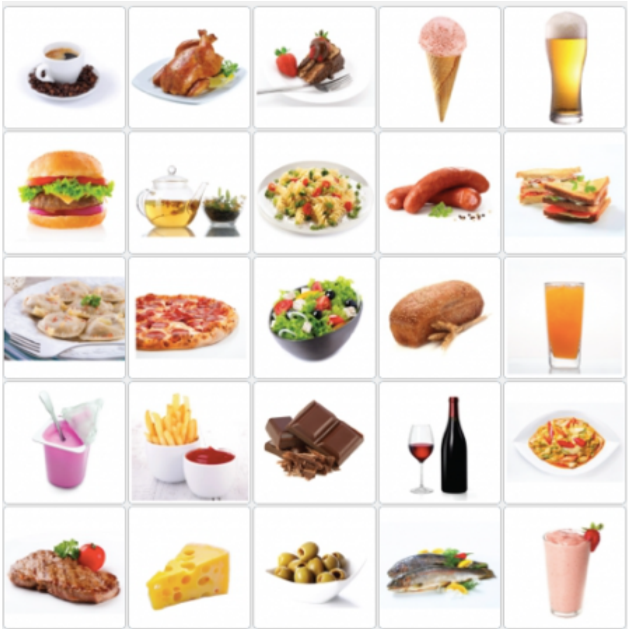

Figure 5. Memory task
Participants are first presented with 5 objects for 5 seconds. Then, objects disappear and a 5 × 5 matrix of objects is presented for 20 seconds. Participants need to find the five objects and remember their location. Next, all objects disappear and participants need to click on boxes in which the five objects were just presented.

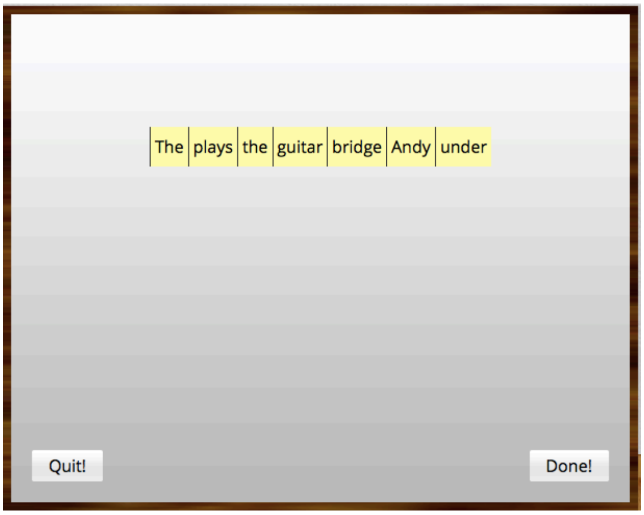

Figure 6. Jumbled sentences
Participant's task is to arrange scrambled words into meaningful sentences. This can be done by clicking on each word with the mouse cursor and dragging it to the right position.

ADDITIONAL STUDY

Participants

The sample comprised fourty-six mTurk users (30 men, 16 women). The age ranged from 21 to 51 years with the mean of $M = 33.52$ ($SD = 7.82$). Participants were given a monetary compensation of $3 for participation in the study.

Measures and Procedure

Measures and procedure was the same as in Study 2 described in the main text. The reliability of the Need for Cognitive Closure scale was equal to $\alpha = .81$ ($M = 4.35$, $SD = 0.64$). Mean score on the goal importance item was equal to $M = 4.04$, $SD = 0.82$.

Results & Discussion

Descriptive statistics for performance are presented in Table 1. To test the effects of NFC and effort investment on task performance, we ran a mediation analysis with the use of the Process macro for SPSS (Hayes, 2013; model 4). The number of completed tasks was controlled for.

Using 10,000 bootstrap samples, we found an indirect effect of NFC on task performance (the number of points earned) through effort investment (time spent per task), $IE = 1.84$, 95% *CI* [0.04, 5.20]. The relationship between NFC and task performance remained non-significant when effort investment was entered into the regression model, $DE = 2.51$, 95% *CI* [−4.97, 5.15]. Thus, the higher NFC, the more time spent per task, which translates into more points earned in the task. The relationship was not dependent on task goal importance (the index of moderated mediation run for model 7 was non-significant and equal to $IMM = 0.35$, $SE = 1.56$, 95% *CI* [-2.73, 3.75]).

Table 1

Descriptive statistics for effort and task performance in the additional study (N = 46).

	M	*SD*
Total points	92.01	22.43
Tasks completed	20.91	4.51
Time per task [s]	85.75	32.04

ADDITIONAL ANALYSES

Here we present additional analyses testing the double mediation model as presented in Figure 7.

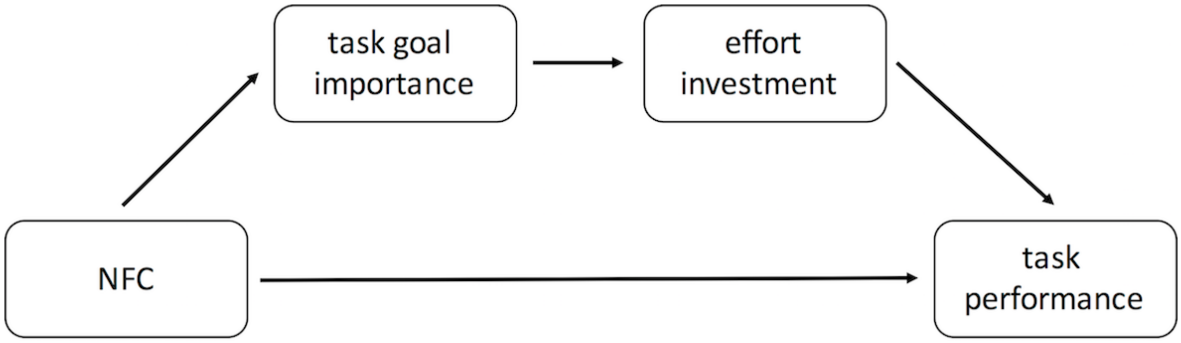

Figure 7. Theoretical model tested in additional analyses.

We predicted that in Study 1 NFC and task goal importance should not be positively related. Therefore, there should be no significant effect of NFC on task performance mediated by goal importance and effort. However, such an effect should be significant in Studies 2 and 3, wherein best task performance is the only (Study 2) or best (Study 3) way to attain closure.

To test these predictions, we run a double mediation analysis (Process macro for SPSS, Hayes, 2013; model 6; 10,000 bootstrap samples). Sample sizes as well as control variables were the same as the ones described for each study in the manuscript.

Study 1

The results showed no significant effect of NFC on goal importance, $b = 0.13$, $SE = 0.09$, $t = 1.37$, $p = .17$, 95% CI [-0.06, 0.32]. Goal importance significantly predicted effort, $b = 12.50$, $SE = 4.31$, $t = 2.90$, $p = .005$, 95% CI [3.95, 21.05], and effort predicted performance, $b = 0.14$, $SE = 0.06$, $t = 2.18$, $p = .03$, 95% CI [0.01, 0.26]. The overall mediation model was not significant, $IE = 0.22$, $SE = 0.19$, 95% CI [-0.01, 0.82].

Study 2

The results showed a significant effect of NFC on goal importance, $b = 0.32$, $SE = 0.11$, $t = 2.97$, $p = .004$, 95% CI [0.11, 0.53]. Goal importance significantly predicted effort, $b = 14.26$, $SE = 3.60$, $t = 3.96$, $p < .001$, 95% CI [7.10, 21.41], and effort predicted performance, $b = 0.18$, $SE = 0.04$, $t = 4.22$, $p < .001$, 95% CI [0.10, 0.27]. The overall mediation model was also significant, $IE = 0.82$, $SE = 0.43$, 95% CI [0.24, 2.12].

Study 3

Like in Study 2, the results showed a significant effect of NFC on goal importance, $b = 0.29$, $SE = 0.14$, $t = 2.04$, $p = .04$, 95% CI [0.01, 0.57]. Goal importance significantly predicted effort, $b = 8.58$, $SE = 4.28$, $t = 2.01$, $p < .05$, 95% CI [0.07, 17.08], and effort predicted performance, $b = 0.11$, $SE = 0.04$, $t = 3.05$, $p = .003$, 95% CI [0.04, 0.19]. The overall mediation model was also significant, $IE = 0.28$, $SE = 0.23$, 95% CI [0.02, 1.00].
